# Paraquat‐induced acute kidney and liver injury: Case report of a survivor from Bangladesh

**DOI:** 10.1002/ccr3.5020

**Published:** 2021-11-06

**Authors:** Md Asaduzzaman, Monotush Ronjon Chando, Nasad Ahmed, Khandaker Mohammad Rezwanul Islam, Munsi Mohammad Jahangir Alam, Soumitra Roy

**Affiliations:** ^1^ Department of Medicine Sylhet M.A.G Osmani Medical College Hospital Sylhet Bangladesh

**Keywords:** acute kidney injury, acute liver injury, paraquat, survivor

## Abstract

Despite high fatality following paraquat ingestion, a few percentages of patients survive even after organ damage appears. We need to focus more on careful clinical and laboratory monitoring. Early diagnosis and Supportive therapy are crucial.

## Graphical abstract

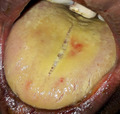

## 1 INTRODUCTION

Mortality following paraquat (PQ) ingestion remains very high. A young boy with PQ ingestion was treated at our center and he survived even after the development of acute kidney and liver injury. Appropriate monitoring of clinical status and laboratory parameters is a crucial part of the management of this poisoning.

Paraquat (PQ, 1,1′‐dimethyl‐4‐4′‐bipyridinium dichloride) is a highly toxic herbicide widely used in agriculture throughout the world, has been marketed for more than 50 years. Despite numerous intoxications, PQ is now registered and used in over 120 developed and developing countries throughout the world.^1^ Deliberate self‐poisoning with paraquat continues to be a major public health concern in many developing countries. The case fatality is very high in all centers despite large variations in treatment^2^, ^3^ and the mortality varies between 50% and 90%, but in cases of intentional self‐poisoning with concentrated formulations, mortality approaches 100%.^2^ Here, we described the scenario of a young boy who presented with a history of PQ ingestion and ultimately survived.

## 2 CASE DESCRIPTION

A young patient of 18 years came to our hospital on May 17, 2021, with a history of PQ (approx 5 ml of “Gramoxone 20 SL”**)** consumption 5 days back. Identification of the poison as paraquat was based on recollection by the patient and examination of the bottle brought alongside. Following ingestion, initially, he was taken to local Upazila hospital where he was given gastric lavage. He did not continue treatment in that hospital as he had no significant complaints. Three days after his return to the house, he started to feel pain in the oral cavity associated with difficulty in swallowing. Subsequently, the patient was admitted to regional tertiary care hospital, Sylhet M.A.G Osmani Medical College Hospital, Sylhet, Bangladesh for better management.

At the time of arrival at our center, the patient was found conscious and well oriented. His presenting complaints were swallowing difficulties and decreased volume of urine for 1 day. He did not complain of shortness of breath, chest pain, or cough. He denied any history of vomiting. On examination, there was burn on the lips. He could not talk properly due to pain in the oral cavity. Anemia, cyanosis, and edema were absent. The patient was dehydrated and icteric. Pulse was 82 beats per minute, blood pressure (BP) 110/70 mmHg, and respiratory rate was 24 breaths per minute. The temperature was 99°F. Peripheral capillary oxygen saturation (SpO_2_) was 97% while breathing room air at the time of admission.

**FIGURE 1:**
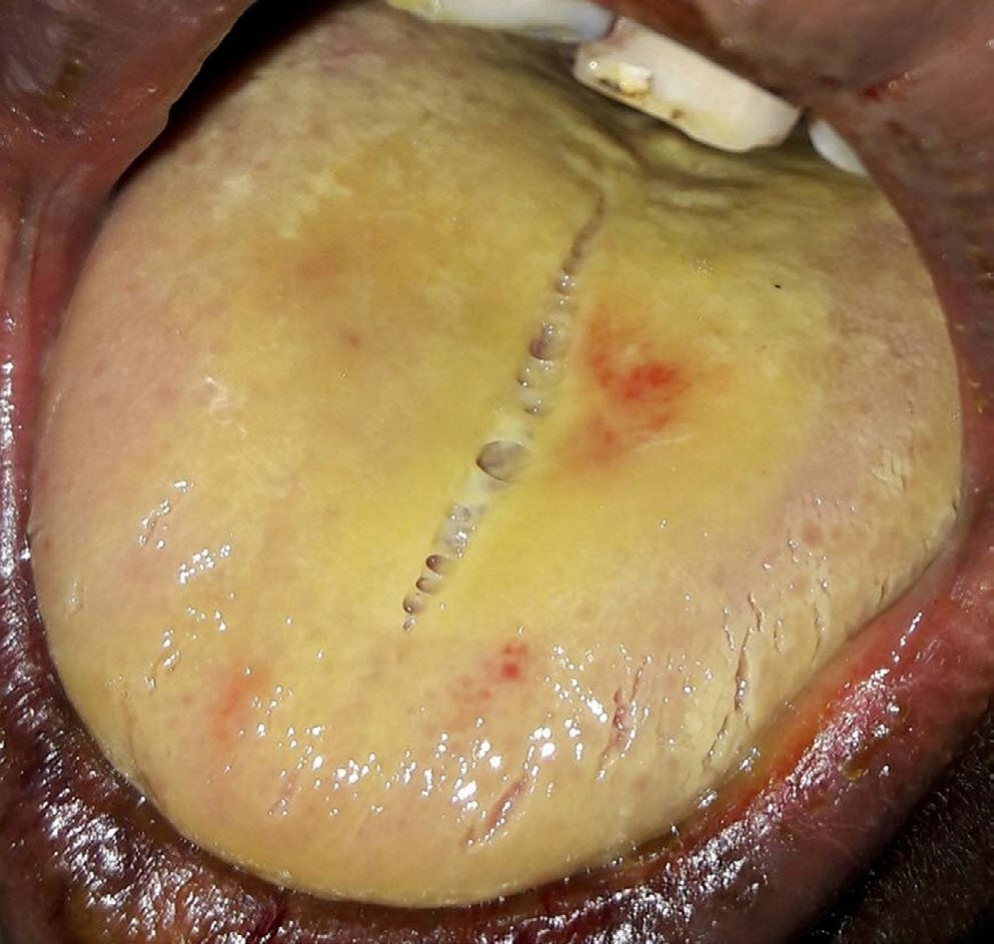
Picture of the tongue of the patient. Tongue coated with yellowish necrotic slough following paraquat ingestion

Pupils were not constricted. Examination of the skin and eyes reveals no evidence of topical contact. Inspection of the oral cavity revealed that the tongue was coated with slough (Figure 1), and there were multiple ulcers in the tongue. Multiple erosions over his lips and oral cavity were also seen. Further details of the oral cavity could not be seen because the patient could not open his oral aperture wide and protrude the tongue due to severe pain. His chest was clear with no crepitations or wheeze. Abdominal examination revealed normal findings except mild tenderness in the epigastrium and right hypochondrium. Other systemic examination was normal. The patient was managed conservatively with nothing per oral, intravenous fluid, and proton pump inhibitor. His vital signs were monitored closely. Throughout the hospital stay his vital signs were within normal limits. Initially, his urine volume was less, about 500 ml on day 1, but as the days go by urine volume increased, and on the last hospital day it was 2200 ml in the last 24 hours. His SpO2 was within the normal range during the hospital stay. On the day of admission, his serum creatinine, blood urea nitrogen (BUN), bilirubin, and alanine transaminase (ALT) levels were high (11.6 mg/dl, 96 mg/dl, 12.8 mg/dl, and 265 IU/L, respectively). Considering the impairment of renal function (Stage 3, acute kidney injury (AKI), according to KDIGO Clinical Practice Guideline,^4^ we decided to give hemodialysis, but the patient could not afford it due to financial constraints. Anyway over the course of the next few days, the level of both (serum creatinine and ALT) dropped down to an almost normal level. Other investigations like complete blood count (CBC), Chest X‐ray, and S. electrolyte were within normal range except mild hypokalemia on one occasion (Table 1).

**TABLE 1:** Laboratory data of the patient

	Day 1 (17.5.21)	Day 2	Day 3	Day 5	Day 8	Day 10
CBC	Hb ‐ 14.9 WBC‐ 5.93×10^9^/L NE‐ 68% LY‐ 24.8% Platelet‐ 262 ×10^9^/L	Not done	Not done	Not done	Not done	Hb‐ 14 WBC−6×10^9^/L NE‐ 70% LY‐ 25% Platelet−310×10^9^/L
S. creatinine (mg/dl)	11.6	8.8	5.73	1.40	0.89	0.80
ALT(IU/L)	265	249	233	175	60	36
Bilirubin (total)(mg/dl)	12.8	Not done	Not done	Not done	Not done	Not done
BUN (mg/dl)	96	Not done	Not done	Not done	Not done	Not done
S. electrolyte	Not done	Not done	Not done	Na‐ 141.9 mmol/L K‐ 2.78 mmol/L CL‐ 100.7 mmol/L CO_2_‐ 29 mmol/L	Not done	Na‐ 140 mmol/L K‐ 4.01 mmol/L CL‐ 101 mmol/L CO_2_‐ 30 mmol/L
Chest X‐ray	Not done	Not done	Not done	No abnormality detected	Not done	No abnormality detected

Abbreviations: ALT, Alanine transaminase; BUN, Blood Urea Nitrogen; Hb, Hemoglobin; LY, Lymphocyte; NE, Neutrophil; WBC, White blood cell.

On the 10th day since admission, he was discharged with advice to attend a follow‐up visit at our center. He attended two follow‐up visits, first at 1 month and second at 2 months, and clinically found to be satisfactory with no swallowing or other difficulties.

## 3 DISCUSSION

Self‐poisoning with pesticides accounts for 14–20% of global suicides, an estimated 110,000–168,000 deaths each year.^5^ This is most prevalent in South Asian countries as these pesticides are easily available and are being used widely in agriculture.^6^ Its fast‐acting nature, stability, availability, and affordability, and the lack of an effective antidote, make PQ an extremely hazardous substance. The most frequent routes of exposure to paraquat, either accidentally or intentionally, in humans and animals are following ingestion or through direct skin contact.

PQ poisoning is less commonly reported in developing countries where cases of organophosphate (OPC) poisoning are frequent. Some cases of PQ poisoning are sometimes treated as a case of OPC poisoning mistakenly which sometimes leaves a detrimental effect on patients. This leads to delayed delivery of appropriate supportive care to these patients which probably contribute to poor outcome in this group of patients.

The exact mechanism of toxicity caused by PQ is not known yet. Following PQ poisoning, the lungs are the main target organs, and the redox reaction occurs after the uptake of PQ in the lungs, which interferes with mitochondrial electron transfer, generates a large number of oxygen free radicals, and induces lipid peroxidation injury.^7^ In the alveolar epithelium, absorbed paraquat concentrations can be up to 10 to 20 times the serum paraquat levels.^8^ Numerous studies found that outcome is related to the plasma concentration of PQ.^9^, ^10^, ^11^, ^12^

The symptomatology of human PQ poisonings can be divided into three different presentations depending on the amount of ingested PQ. Ingestion of PQ ion of less than 20–30 mg/kg produces no symptoms or only mild GIT symptoms (nausea, irritation, and diarrhea). Patients who ingest >20–30 but <40–50 mg/kg are most likely to die from pulmonary fibrosis, which progresses after a few days to a few weeks. Patients who ingest greater than 40 mg/kg (>15 ml of a 20% solution for a 70‐kg patient) usually die within hours to a few days, at most. These patients experience multiple organ failures, including acute respiratory distress syndrome (ARDS), cerebral edema, myocardial necrosis, with cardiac, neurologic, adrenal, pancreatic, hepatic (with jaundice), and renal failure.^13^, ^14^ The first toxicological effects to the lung correspond to a destructive phase in which the alveolar type I and type II epithelial cells are destroyed. This occurs within 1–3 days of dosing, although the speed at which it occurs depends on the given dose and the route of administration. The second phase of PQ‐induced lung toxicity involves the development of extensive fibrosis in the lung, which is probably a compensatory repair mechanism to the damaged alveolar epithelial cells.^1^ If the degree of lung exposure to PQ is high, the alveolitis will be more widespread and severe, thereby resulting in more extensive fibrosis and severe anoxia. Surprisingly our patient did not have obvious clinical evidence of lung injury as evidenced by the absence of dyspnea, tachypnea, cough, chest pain, and hypoxia. The reason is not obvious.

The mechanism whereby PQ causes AKI is not fully understood; however, it is known that this compound can accrue within renal tubular cells, leading to cycles of reduction and oxidation, generating reactive oxygen species, and ultimately damaging the proximal tubules.^15^ As the kidney is the main organ responsible for paraquat excretion, the resultant kidney injury may reduce the elimination of paraquat and increase its toxicity in other organs.^16^ Anyway, kidney injury may occur due to volume depletion resulting from inadequate fluid intake. In our case, the renal impairment may be due to a combination of PQ‐induced tubular damage and pre‐renal AKI caused by poor oral intake due to odynophagia. But a low BUN/Creatinine ratio (8.27) hints at renal damage as a cause of AKI in our case.

The liver is the main source of intrinsic antioxidants that play an important role in enzymatic metabolism and detoxification. Therefore, the liver is more vulnerable to ROS‐mediated injury. Previous studies show that PQ intoxication results in acute liver injury characterized by persistent elevation of liver aminotransferases and histopathological changes.^2^, ^17^, ^18^ An observational study involving 187 cases of intentional PQ ingestion found a high prevalence of toxic hepatitis (46.52%) and greater incidences of acute respiratory failure and acute renal failure in this group of patients than patients without hepatitis.^19^ Our patient also suffered from toxic hepatitis.

The management of PQ poisoning is mostly supportive as there is no antidote available to date. Gastric lavage, activated charcoal, and Fuller's Earth if initiated early can alleviate organ damage and improve survival.^20^, ^21^, ^22^ A recent meta‐analysis showed that hemoperfusion combined with continuous veno‐venous hemofiltration can reduce the short‐term mortality and the incidence of circulatory failure, prolong the survival time but no significant impact long‐term prognosis.^23^ The survival rate was better in patients who received early (<6 hours) hemoperfusion.^24^ Different antioxidants in alone or in combination have been shown to reduce PQ‐induced organ damage.^25^, ^26^, ^27^, ^28^ A recent Cochrane Review concluded that there is low‐certainly evidence that glucocorticoids with cyclophosphamide in addition to standard care may slightly reduce mortality in hospitalized people with oral paraquat poisoning and may have little or no effect on mortality at three months after hospital discharge.^29^ In the present case study, we managed the case with only supportive treatment without using any immunosuppressive drug or antioxidants given their uncertainty of evidence.

Young age, percutaneous or inhalational route, exposure to less paraquat, and lesser degrees of leukocytosis, acidosis, and renal, hepatic, and pancreatic failures on admission are predictors of survival after acute PQ poisoning.^30^ Our reported patient survived probably due to his younger age, early application of gastric lavage, absence of leukocytosis, and no significant impairment of lung function. We suggest frequent monitoring of clinical status and laboratory parameters and best supportive management to be given in all cases of PQ poisoning.

## 4 CONCLUSION

As there is no specific antidote to paraquat poisoning currently available; hence, an early aggressive gastrointestinal decontamination should get priority in cases of PQ poisoning. An early diagnosis and a close look at vital parameters and dynamic changes in laboratory parameters can help in better understanding of disease course and can guide the clinician to provide appropriate supportive management. Public awareness should be raised on PQ toxicity and the Government should impose a ban on this herbicide.

## CONFLICT OF INTEREST

The authors declare no conflict of interest.

## AUTHOR CONTRIBUTIONS

MA made substantial contributions to conception and design, acquisition and interpretation of data, drafting the manuscript, and revising it critically for important intellectual content. MRC, NA, KMRI, MMJA, and SR diagnosed the case and made substantial contributions to acquisition and interpretation of data and revising the manuscript critically for important intellectual content. All authors were directly involved in the management of this patient, read and approved the final manuscript, and agreed to be accountable for all aspects of the work in ensuring that questions related to the accuracy or integrity of any part of the work are appropriately investigated and resolved.

## ETHICAL APPROVAL

This study was carried out following the recommendations of the ethics committee of the Sylhet M.A.G Osmani Medical College Hospital, Sylhet 3100, Bangladesh.

## CONSENT

Written informed consent was obtained from the patient for the publication of this case report.
